# The participation of activating transcription factor 6 alpha, ATF6α, in Alzheimer’s disease

**DOI:** 10.1002/alz.083626

**Published:** 2025-01-03

**Authors:** Mariana Gomes Chauvet, Alinny Rosendo Isaac, Mychael V Lourenco

**Affiliations:** ^1^ Federal University of Rio de Janeiro, Rio de Janeiro, Rio de Janeiro Brazil; ^2^ Federal University of Rio de Janeiro, Rio de Janeiro Brazil; ^3^ Institute of Medical Biochemistry Leopoldo de Meis, Federal University of Rio de Janeiro, Rio De Janeiro, Rio de Janeiro Brazil

## Abstract

**Background:**

Alzheimer’s disease (AD) is the leading cause of dementia in elderly humans worldwide. More than 40 million people currently suffer from AD, and this prevalence tends to increase considerably in the coming decades due to increased longevity. The unfolded protein response (UPR) is an adaptive signaling mechanism that aims to maintain cell viability under misfolded protein accumulation and endoplasmic reticulum stress. UPR activation is associated with neurodegenerative diseases, such as AD, leading to synaptic loss and neuronal death. A key but understudied branch of the UPR depends on the activation of transcription factor 6 α (ATF6α). ATF6α was recently proposed as a therapeutical target in heart disease. Nonetheless, the association between ATF6α signaling and AD remains elusive. Here, we investigated whether dysfunctional ATF6α associates with AD, using both human *post‐mortem* brain tissues and mouse models.

**Method:**

ATF6α protein levels were measured in *post‐mortem* cerebral cortex from healthy controls (HC) and AD patients, as well as in the hippocampus of APP/PS1 transgenic mice. Using an online database (Aging, dementia and TBI study from the Allen Institute) ATF6α mRNA expression was analyzed in HC and AD subjects.

**Result:**

Initial results suggest that ATF6α protein levels are reduced in AD patients. Using the Allen Institute database, we also found reduced ATF6α mRNA expression, in relation to the Braak scale of tau pathology in the parietal neocortex and hippocampus. Conversely, we found increased ATF6α protein levels in the hippocampus of the APP/PS1 transgenic mouse model of amyloid pathology.

**Conclusion:**

Together, our results indicate that ATF6α may be differentially altered in AD. Further investigation of the ATF6α pathway in AD may offer a novel therapeutic perspective for cognitive decline.

